# Sleep Phenotypes, Genetic Susceptibility, and Risk of Obesity in Patients With Type 2 Diabetes: A National Prospective Cohort Study

**DOI:** 10.1111/1753-0407.70095

**Published:** 2025-05-20

**Authors:** Lei Xi, Li Li, Songbo Fu, Yuancheng Dai, Juan Shi, Yanmei Yu, Ying Peng, Hongmei Qiu, Jinsong Kuang, Hongyun Lu, Huige Shao, Chunlei Yuan, Xiaohu Wang, Ping Zhang, Sheli Li, Yanhui Pan, Ling Hu, Zhigang Zhao, Yunxia Chen, Jian Kuang, Yi Shu, Jinhua Qian, Qibin Mao, Jieji Zhang, Yan Liu, Hong Yang, Zhaoli Yan, Weici Xie, Qian Zhang, Ping Zhang, Hongji Wu, Ling Gao, Yongjun Jin, Ning Xu, Chaoyang Xu, Xiaohui Sun, Zhimin Feng, Qing Zhang, Lin Li, Guang Ning, Yifei Zhang, Yanan Cao, Weiqing Wang

**Affiliations:** ^1^ Department of Endocrine and Metabolic Diseases, Shanghai Institute of Endocrine and Metabolic Diseases, National Clinical Research Center for Metabolic Diseases (Shanghai), Ruijin Yangtze River Delta Health Institute, Research Unit of Clinical and Basic Research on Metabolic Diseases of Chinese Academy of Medical Sciences, Wuxi Branch of Ruijin Hospital, Ruijin Hospital Shanghai Jiao Tong University School of Medicine Shanghai China; ^2^ Department of Endocrinology The First Affiliated Hospital of Ningbo University Ningbo China; ^3^ Department of Endocrinology The First Hospital of Lanzhou University Lanzhou China; ^4^ Department of Internal Medicine of Traditional Chinese Medicine Sheyang Diabetes Hospital Yancheng China; ^5^ Department of Endocrinology Mudanjiang Cardiovascular Hospital Mudanjiang China; ^6^ Department of Endocrinology and Metabolism People's Hospital of Yuxi City (The Sixth Affiliated Hospital of Kunming Medical University) Yuxi China; ^7^ Department of Endocrinology and Metabolism Shenyang Fourth People Hospital Shenyang China; ^8^ Department of Endocrinology and Metabolism, Zhuhai Clinical Medical College Affiliated With Jinan University Zhuhai People's Hospital Zhuhai China; ^9^ Department of Endocrinology Changsha Central Hospital Changsha China; ^10^ Department of Endocrinology Shandong Health Zibo Hospital Zibo China; ^11^ Department of Endocrinology and Metabolism Anyang People's Hospital Anyang China; ^12^ Department of Endocrinology The First People's Hospital of YuLin Yulin China; ^13^ Department of Endocrinology and Metabolism Yan'an University Affiliated Hospital Yan'an China; ^14^ Department of Endocrinology Qixia City People's Hospital Qixia China; ^15^ Department of Endocrinology The Third Affiliated Hospital of Nanchang University (The First Hospital of Nanchang) Nanchang China; ^16^ Department of Endocrinology Yihe Hospital Zhengzhou China; ^17^ Department of Endocrinology and Metabolism Cangzhou People's Hospital Cangzhou China; ^18^ Department of Endocrinology, Guangdong Provincial People's Hospital Guangdong Academy of Medical Sciences Guangzhou China; ^19^ Department of Endocrinology, The Sixth Affiliated Hospital, School of Medicine South China University of Technology Foshan China; ^20^ Department of Endocrine and Metabolic Diseases The People's Hospital of Pizhou Pizhou China; ^21^ Department of Endocrinology and Metabolism Shaoxing Second Hospital Shaoxing China; ^22^ Department of Endocrinology Fenghua District Traditional Chinese Medicine Hospital of Ningbo Ningbo China; ^23^ Department of Endocrinology The Third People's Hospital of Datong Datong China; ^24^ Department of Endocrinology Ruian People's Hospital Ruian China; ^25^ Department of Endocrinology The Affiliated Hospital of Inner Mongolia Medical University Hohhot China; ^26^ Department of Endocrinology The No. 1 General Hospital in Tian Men Tianmen China; ^27^ Department of Endocrinology The Second Affiliated Hospital of Guizhou Medical University Kaili China; ^28^ Department of Endocrinology The Second Affiliated Hospital of Dalian Medical University Dalian China; ^29^ Department of Endocrinology People's Hospital of Putuo District Zhoushan China; ^30^ Department of Endocrinology, Xiangyang Central Hospital Affiliated Hospital of Hubei University of Arts and Science Xiangyang China; ^31^ Department of Endocrinology and Metabolism Yantai Affiliated Hospital of Binzhou Medical University Yantai China; ^32^ Department of Endocrinology The First People's Hospital of Lianyungang Lianyungang China; ^33^ Department of Endocrinology Jiangsu Shengze Hospital Suzhou China; ^34^ National Center for Translational Medicine (Shanghai), Institute of Translational Medicine Shanghai Jiao Tong University Shanghai China

**Keywords:** genetic risk, obesity, sleep, type 2 diabetes, weight gain

## Abstract

**Background:**

To determine the associations between sleep phenotypes and the risks of specific obesity types and weight gain in patients with type 2 diabetes (T2D), especially in different genetic risk groups.

**Materials and Methods:**

We conducted a prospective study involving 58 890 participants. Sleep and napping were assessed according to the standardized questionnaire. General and abdominal obesity were defined by BMI or visceral fat area (VFA), respectively. Multivariable Cox regression, stratified, and joint analysis were performed to explore potential correlations. Furthermore, mediation models were constructed to figure out the mediating role of metabolic factors (blood pressure, UACR, and HbA1c).

**Results:**

During a median 3.05‐year follow‐up period, short sleep increased the risk of obesity (HR 1.42, 95% CI 1.17–1.71; 1.33, 1.08–1.65) and weight gain (1.21, 1.09–1.34; 1.17, 1.06–1.29), while long sleep and napping were unrelated to abdominal obesity and weight gain. Mediation analysis showed that systolic blood pressure, UACR, and HbA1c mediated the statistical association between night sleep duration and general obesity with proportions (%) of 7.9, 1.8, and 8.8, respectively. Joint analysis showed both sleep and napping groups had no significance among the low genetic risk group, while long napping, short sleep, and long sleep increased the risk of general obesity in medium to high risk patients.

**Conclusions:**

Short sleep, long sleep, and long napping increased the risk of general obesity and BMI‐defined weight gain, and were more pronounced in the medium to high genetic risk group. Napping was unrelated to abdominal obesity. Metabolic factors partially explain the mechanism between sleep and obesity.

## Graphical abstract

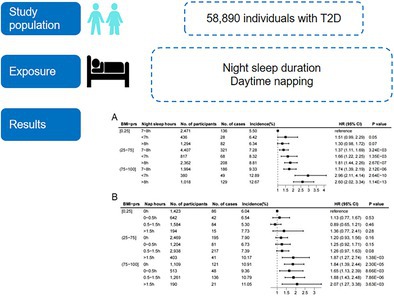

**Summary**

- Night sleep duration, but not naps, increased the risk of abdominal obesity.
- Short sleep (< 7 h), long sleep (> 8 h), and long napping (> 1.5 h) increase the risk of general obesity.
- The above conclusions were more pronounced in the medium to high genetic risk group of obesity.
- Metabolic factors partially explain the mechanism between sleep and obesity.

## 1 Introduction

Diabetes and obesity are serious global health problems. According to data from the World Health Organization, there are over 1.9 billion people with overweight worldwide, of which 650 million are with obesity. At the same time, more than 400 million people worldwide suffer from diabetes, and the number is still rising [1, 2]. Obesity and diabetes, especially type 2 diabetes (T2D), are closely related. Many patients with T2D have insulin resistance, which can also affect fat metabolism, making it easier for fat to accumulate in the body, leading to obesity [3, 4, 5].

T2D is a disease closely related to lifestyle and genetic factors. Sleep, as an important part of lifestyle, has received increasing attention in recent years regarding its impact on health [5, 6]. According to the 2024 China Sleep Research Report [7], the night sleep duration of Chinese residents has decreased by 1.5 h compared to 10 years ago, with an average sleep duration of 7.37 h. Among them, 29.4% of respondents had less than 7 h of sleep and generally had poor sleep quality, with over 300 million people experiencing sleep disorders. Adjusting the length of different sleep phenotypes, including night sleep and nap, can improve the long‐term prognosis of cardiovascular diseases and Alzheimer's disease. However, their impact on obesity and weight gain is currently inconclusive, especially in patients with T2D. Furthermore, Whether metabolic factors such as blood pressure, HbA1c, and urinary albumin‐to‐creatinine ratio (UACR) play a role in it is still unclear [8, 9, 10, 11].

Based on these observations, we hypothesized that night sleep and napping duration can influence the obesity status in patients with T2D, and that the phenomenon was influenced by genetic and metabolic factors. Therefore, based on research data of 72 652 adult people with T2D from the 200 diabetes centers known as the Metabolic Management Centers (MMCs), we aimed to assess the associations of specific obesity types, weight gain with night sleep duration and daytime napping behavior after taking into account multivariate adjustment. In addition, we aimed to examine whether there were heterogeneous associations between different genetic susceptibility groups and to identify mediators of metabolic factors (blood pressure, UACR, and HbA1c).

## 2 Methods

### 2.1 Study Population

The data were derived from MMCs, an innovation project for the management of metabolic diseases and complications in China, and detailed information on the MMC program was previously published (Clinical Trials. gov number: NCT03811470) [12, 13]. In this study, 72 652 adult people with T2D were recruited from June 2017 to June 2021, and the last follow‐up data was collected until July 2023. After excluding missing or implausible values of sleep duration (i.e., < 3 or > 12 h/night), use of sleeping aids, or psychiatric medications, 58 890 were finally available for this analysis (Figure S1). The study was approved by the Ethics Committee of Ruijin Hospital and other centers (if essential) and conducted following the Declaration of Helsinki. Informed consent was obtained from all the patients.

### 2.2 Covariate and Exposure Assessment

Data collection was performed in local MMCs by trained interviewers according to a standard protocol at baseline and the follow‐up visit. Detailed information on demographic characteristics, lifestyle factors, and medical records was obtained. Anthropometric measurements were performed in local MMCs, and abdominal visceral fat area (VFA) was measured by bioelectrical impedance technology. Standard protocols for collecting and handling blood and urine samples have been described in our previous study [14, 15].

Age, sex, systolic blood pressure (SBP), diastolic blood pressure (DBP), body mass index (BMI), and duration of diabetes were gathered based on the MMC system. Ideal smoking was defined as a person who never smoked or quit smoking > 12 months. Nondrinker was defined as a person who never drank alcohol or quit. The UACR was derived by calculating the ratio between the concentration of albumin and creatinine in the urine. Ethnic groups include Han and minority nationalities. Diabetes duration was defined as the years between the first diagnosis of diabetes and the MMCs baseline assessment for diagnosed patients or 0 years for newly diagnosed patients.

Habitual night sleep duration and daytime napping duration were self‐reported through the baseline questionnaire with the following question: (1) “How many hours do you sleep every night (Record the time of falling asleep and waking up)?”; (2) “How many hours do you take a daytime napping every day at noon?” Time recording adopts a 24‐h system and the answer to this question is accurate to the minute and all results of sleep duration have been converted into hours (e.g., 7 h 30 min = 7.5 h). We stratified night sleep duration into short (< 7 h), intermediate (7–8 h), and long (> 8 h) groups; daytime napping duration was divided into no nap (0 h), short (> 0–0.5 h), intermediate (> 0.5–1.5 h), and long (> 1.5 h) groups. 7–8 h of night sleep and no nap were used as references [9, 16].

### 2.3 Outcome Assessment

Type of obesity included general obesity and abdominal obesity. General obesity was defined as a BMI of 28 kg/m^2^ or greater according to the criteria of the National Bureau of Disease Control and Prevention of China [17, 18] and abdominal obesity was defined as a VFA of 119.5 cm^2^ or greater, which was in the highest quartile of the study population. In follow‐up analysis, an increase of more than 5% in BMI or VFA from baseline was defined as weight gain.

### 2.4 Generation of Polygenic Risk Score for BMI

Polygenic risk score for BMI (BMI‐PRS, PGS catalog accession ID: PGS002360) was retrieved from the Polygenic Score (PGS) Catalog, which was founded in 2019 by researchers at the University of Cambridge UK, European Bioinformatics Institute, and Baker Institute and aimed to provide an open database of PGS and relevant metadata (http://www.pgscatalog.org). Briefly, development samples included 124 000 East Asian and 920 920 genetic variants from Biobank Japan training data that were identified to construct BMI‐PRS, respectively (Table S1) [19]. BMI‐PRS was calculated by summing the risk alleles, weighted by the effect size derived from the GWAS results. The quality control of individual genotyping data in our study was performed for BMI‐PRS calculation with the criteria including SNP call rate > 0.99, minor allele frequency > 1%, Hardy–Weinberg Equilibrium *p* > 1 × 10^−10^, imputation INFO score ≥ 0.8, sample call rate > 0.98, sample heterozygosity ≤ 3 SD. Detailed methods of data processing, quality control, and analysis were described in our previous studies [20, 21].

### 2.5 Statistical Analysis

The baseline characteristics of the participants were separated by night sleep duration. Cox proportional hazards models were applied to examine the associations of night sleep duration and daytime napping with the risk of general obesity, abdominal obesity, and weight gain, using night sleep duration of 7–8 h and no nap as the reference. Hazard ratios (HRs) with 95% CIs were estimated from two models: In a minimally adjusted model, we adjusted for age and sex as covariates. The primary model further adjusted for ethnicity, smoking, alcohol consumption, SBP, DBP, UACR, HbA1c, and duration of diabetes.

The joint association of different sleep phenotypes and genetic susceptibility with obesity was performed. BMI‐PRS was assessed as a categorical variable, categorized into three groups based on quartiles of PRS values, namely high (0%–25%), moderate (26%–75%), and low genetic risk (76%–100%). Based on different sleep phenotype groups, we compared HRs for participants at moderate and high genetic risk with those at low risk, adjusted for multivariate. Mediation analysis was tested with the metabolic factors (SBP, DBP, UACR, and HbA1c) that showed potential correlations with night sleep duration or daytime napping and obesity, controlling for all confounding factors in the primary model. The quasi‐Bayesian Monte Carlo method with 5000 simulations was adopted to obtain the estimates for the average causal mediation effect, average direct effect, and total effect of potential mediators with the R package “mediation” All statistical analyses were conducted using R software (version 4.1.2). A bilateral test, *p* < 0.05, indicated statistical significance.

## 3 Results

Baseline characteristics are shown in Table 1. A total of 58 890 participants (mean [SD] age, 54.5 [11.2] years; 40.2% women; 96.0% Han Chinese ethnicity) were included in the analysis. The mean (SD) night sleep duration was 7.9 (1.2) h, and 14.1%, 52.8%, and 33.1% reported night sleeping for < 7, 7–8, and > 8 h, respectively. Night sleep duration ranged from 3 to 12 h (median 8 h). Individuals who habitually had a short night sleep duration (5.9 [0.7] h) were more likely to be male (62.8%), younger (54.0 [10.9] years), and have obesity (general obesity 26.4%, abdominal obesity 27.3%), were less likely to have ideal smoking status (69.8%), and be nondrinkers (85.8%), and no daytime napping (37.2%).

**TABLE 1:** Characteristics of the participants at baseline.

	Night sleep duration			
Variables	Total	< 7 h	7–8 h	> 8 h
No.	58 890	8328	31 094	19 468
Age, years	54.5 ± 11.2	54.0 ± 10.9	54.2 ± 11.2	55.3 ± 11.4
Women, *n* (%)	23 646 (40.2)	3094 (37.2)	12 096 (38.9)	8456 (43.4)
Han Chinese ethnicity, *n* (%)	52 719 (96.0)	7291 (95.8)	27 740 (95.9)	17 688 (96.3)
Family history of diabetes, *n* (%)	24 634 (44.2)	3804 (48.8)	13 018 (44.3)	7812 (42.1)
Ideal smoking status, *n* (%)	43 758 (74.6)	5793 (69.8)	22 984 (74.2)	14 981 (77.3)
Nondrinker, *n* (%)	52 060 (88.7)	7131 (85.8)	27 475 (88.6)	17 454 (90.1)
Systolic blood pressure, mmHg	131.6 ± 18.7	131.1 ± 18.2	131.4 ± 18.4	132.2 ± 19.3
Diastolic blood pressure, mmHg	77.2 ± 11.3	77.3 ± 11.3	77.3 ± 11.2	76.9 ± 11.3
Body mass index, kg/m^2^	25.7 ± 3.7	26.1 ± 3.9	25.7 ± 3.7	25.5 ± 3.7
Visceral fat area, cm^2^	96.0 ± 41.6	98.2 ± 42.6	96.2 ± 41.3	94.8 ± 41.5
Night sleep duration, hours/day	7.9 ± 1.2	5.9 ± 0.7	7.6 ± 0.5	9.2 ± 0.8
Daytime napping, hours/day				
None	17 514 (33.2)	2805 (37.2)	8799 (31.7)	5910 (33.8)
> 0–0.5	9958 (18.9)	1429 (19.0)	5503 (19.8)	3026 (17.3)
> 0.5–1.5	21 346 (40.5)	2618 (34.7)	11 749 (42.4)	6979 (39.9)
> 1.5	3935 (7.5)	688 (9.1)	1688 (6.1)	1559 (8.9)
Duration of diabetes, years	7.0 ± 6.9	6.9 ± 6.9	6.9 ± 6.8	7.2 ± 7.0
HbA1c, %	9.0 ± 2.3	9.0 ± 2.3	8.9 ± 2.3	9.1 ± 2.3
HbA1c, mmol/mol	74.8 ± 24.9	74.5 ± 24.7	74.2 ± 24.9	76.0 ± 25.0
UACR, mg/mmol	13.8 ± 71.7	13.6 ± 69.6	12.3 ± 66.0	16.4 ± 80.7
General obesity, *n* (%)	13 697 (23.3)	2191 (26.4)	7217 (23.3)	4289 (22.1)
Abdominal obesity, *n* (%)	12 742 (25.2)	1981 (27.3)	6684 (25.1)	4077 (24.4)

*Note:* Data are mean ± SD or *n* (%).

Abbreviation: UACR Urinary albumin‐to‐creatinine ratio.

Associations of different sleep phenotypes and obesity types are displayed in Table 2. In the clinical analysis, the median (IQR) follow‐up time was 3.05 (1.95–3.84) years. When focusing on the association of night sleep duration with obesity, we documented 1207 incident general obesity cases and 954 incident abdominal obesity cases after excluding participants with general or abdominal obesity at baseline. Compared with participants with intermediate sleep, participants with short sleep had the highest incidence of general obesity (HR, 1.42, 95% CI, 1.17–1.71) and abdominal obesity (1.33, 1.08–1.65), while the relationship between participants with long sleep and different types of obesity was not consistent (general obesity, 1.35, 1.19–1.54; abdominal obesity, 1.11, 0.95–1.29). When focusing on the association of daytime napping with obesity, we documented 1087 incident general obesity cases and 871 incident abdominal obesity cases during the follow‐up. Compared with participants without naps, napping > 1.5 h was positively associated with the incidence of general obesity (1.39, 1.08–1.79) and was not significantly associated with abdominal obesity (1.17, 0.86–1.60). In addition, neither short nap (general obesity, 1.02, 0.84–1.22; abdominal obesity, 0.93, 0.76–1.14) nor intermediate nap (general obesity, 1.01, 0.87–1.16; abdominal obesity, 0.98, 0.83–1.15) was significantly associated with obesity outcomes. Further stratified multivariate analysis focused on the association of night sleep and naps with specific obesity types according to sex and age (Tables S2 and S3). When the outcome was general obesity, a similar pattern of association was observed between the subgroups of age < 60 years and men. However, short sleep and long naps were not associated with general obesity in the subgroups of age ≥ 60 years and women. When the outcome was abdominal obesity, napping, and abdominal obesity were still unrelated in different age and sex groups.

**TABLE 2:** Associations of different sleep phenotypes with obesity types among people with type 2 diabetes.

	General obesity		Abdominal obesity	
Variables	HR (95% CI)	*p* value	HR (95% CI)	*p* value
Night sleep duration				
Intermediate sleep				
Short sleep	1.42 (1.17–1.71)	< 0.01	1.33 (1.08–1.65)	< 0.01
Long sleep	1.35 (1.19–1.54)	< 0.01	1.11 (0.95–1.29)	0.19
Daytime napping				
None				
> 0–0.5	1.02 (0.84–1.22)	0.87	0.93 (0.76–1.14)	0.47
> 0.5–1.5	1.01 (0.87–1.16)	0.92	0.98 (0.83–1.15)	0.76
> 1.5	1.39 (1.08–1.79)	0.01	1.17 (0.86–1.60)	0.31

Abbreviations: HR, hazard ratio; 95% CI, 95% confidence interval.

**FIGURE 1:**
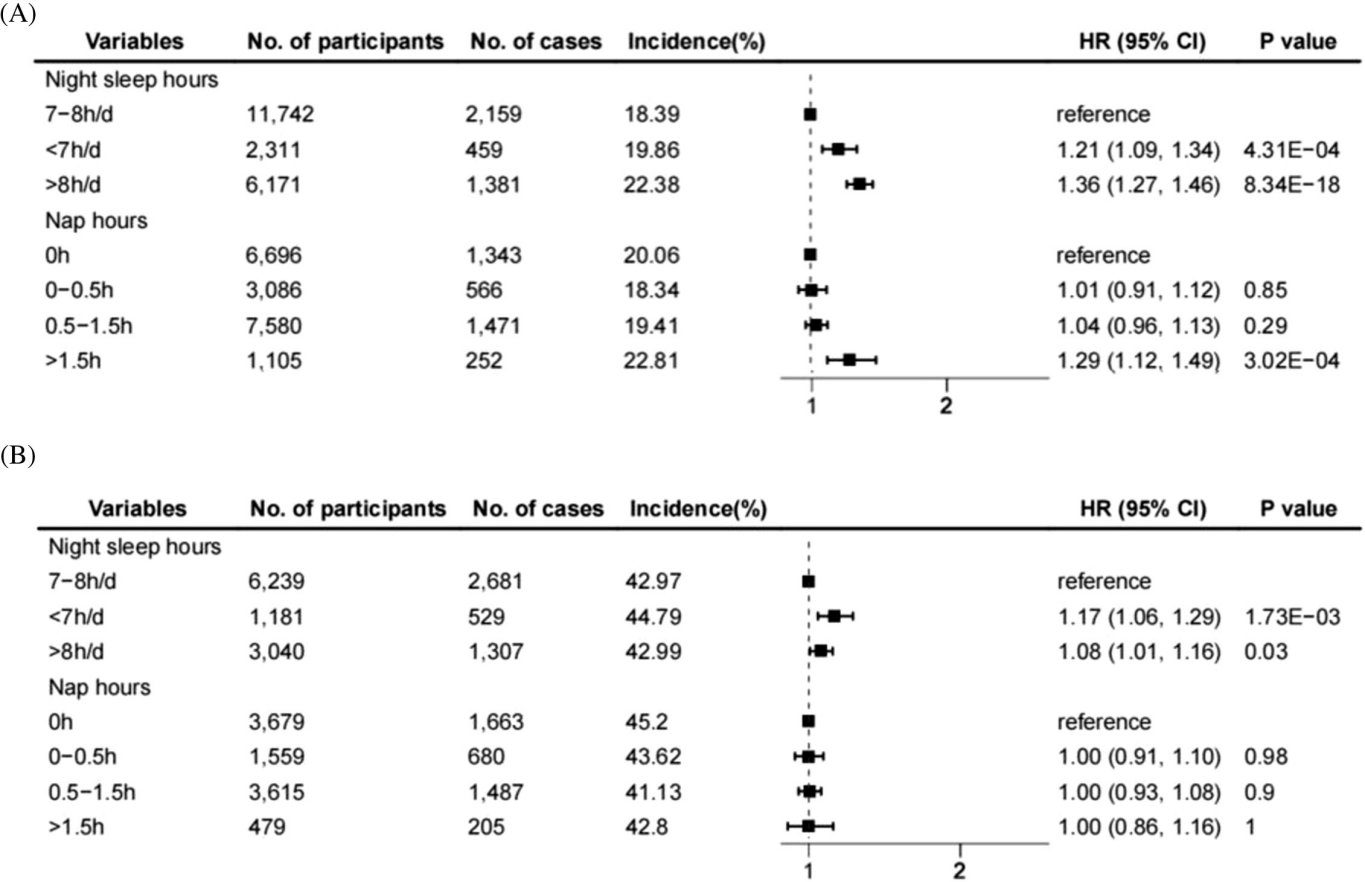
Associations of different sleep phenotypes with weight gain among patients with type 2 diabetes. Weight gain was defined as an increase in BMI (A) or VFA (B) by more than 5%.

Associations of different sleep phenotypes and weight gain are displayed in Figure 1. When weight gain was defined as an increase in BMI by more than 5% during the follow‐up, short sleep (1.21, 1.09–1.34), long sleep (1.36, 1.27–1.46), or long napping (> 1.5 h, 1.29, 1.12–1.49) was positively associated with the incidence of weight gain, respectively. Long and short sleep (1.08, 1.01–1.16; 1.17, 1.06–1.29) exhibited a similar positive effect when involving VFA‐defined weight gain, while nap time had no significant effect on weight gain. Moreover, the above results showed the same trend in the minimally adjusted model (Figure S2 and Table S4).

**FIGURE 2:**
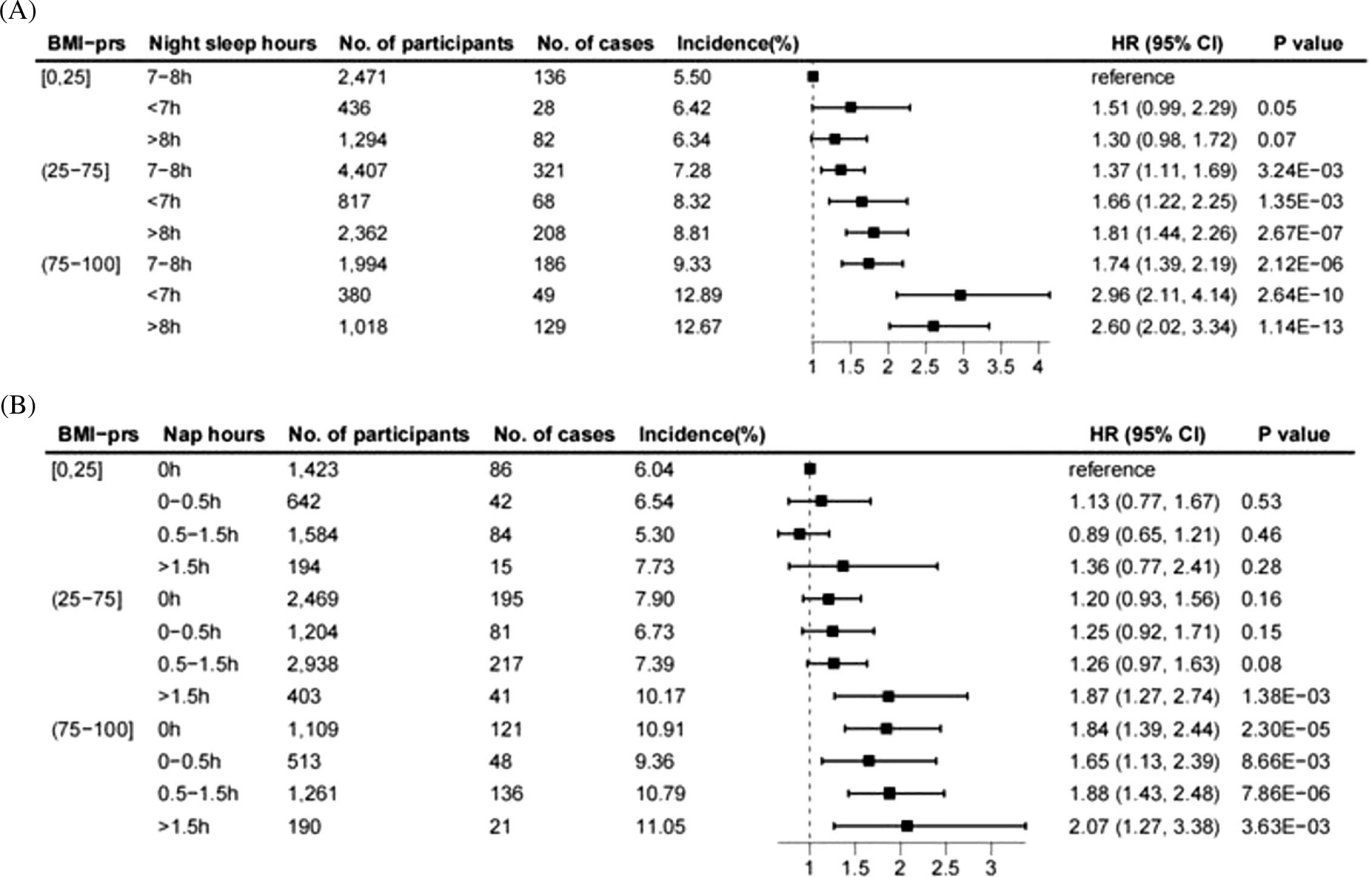
The joint association of night sleep duration (A), nap duration (B), and genetic risk of BMI with general obesity. The polygenic risk score for BMI (BMI‐PRS) was ranked from low to high.

Cox regression results of joint analysis showed that with the increased genetic risk of BMI, the risk of general obesity in T2D patients increased in general (Figure 2). Short sleep, long sleep, and long naps increased the risk of general obesity in patients with T2D, and the phenomenon was more pronounced in the moderate and high genetic risk groups. Specifically, compared with patients with low genetic risk and intermediate sleep, there was no significant change in general obesity risk among patients with short (1.51, 0.99–2.29) and long sleep (1.30, 0.98–1.72) in the low genetic risk group. Compared with patients with low genetic risk and intermediate sleep, all groups of patients with moderate and high genetic risk had an increased risk of general obesity, and the values of HR were more significant in the short and long sleep groups (moderate genetic risk groups, intermediate sleep, 1.37, 1.11–1.69; short sleep, 1.66, 1.22–2.25; long sleep, 1.81, 1.44–2.26; high genetic risk groups, intermediate sleep, 1.74, 1.39–2.19; short sleep, 2.96, 2.11–4.14; long sleep, 2.60, 2.02–3.34). When focusing on the association of daytime napping with general obesity, compared with patients with low genetic risk and no naps, the general obesity risk in the other groups with low genetic risk was not significant. In the moderate genetic risk group, patients with long naps had an increased risk of general obesity (1.87, 1.27–2.74), while the remaining results were not significant. All patients in the high genetic risk group had an increased risk of general obesity, and the HR value was more significant in the long nap group (no nap, 1.84, 1.39–2.44; short nap, 1.65, 1.13–2.39; intermediate nap, 1.88, 1.43–2.48; long nap, 2.07, 1.27–3.38). Similarly, we observed similar changes in the minimally adjusted model (Figure S3).

**FIGURE 3:**
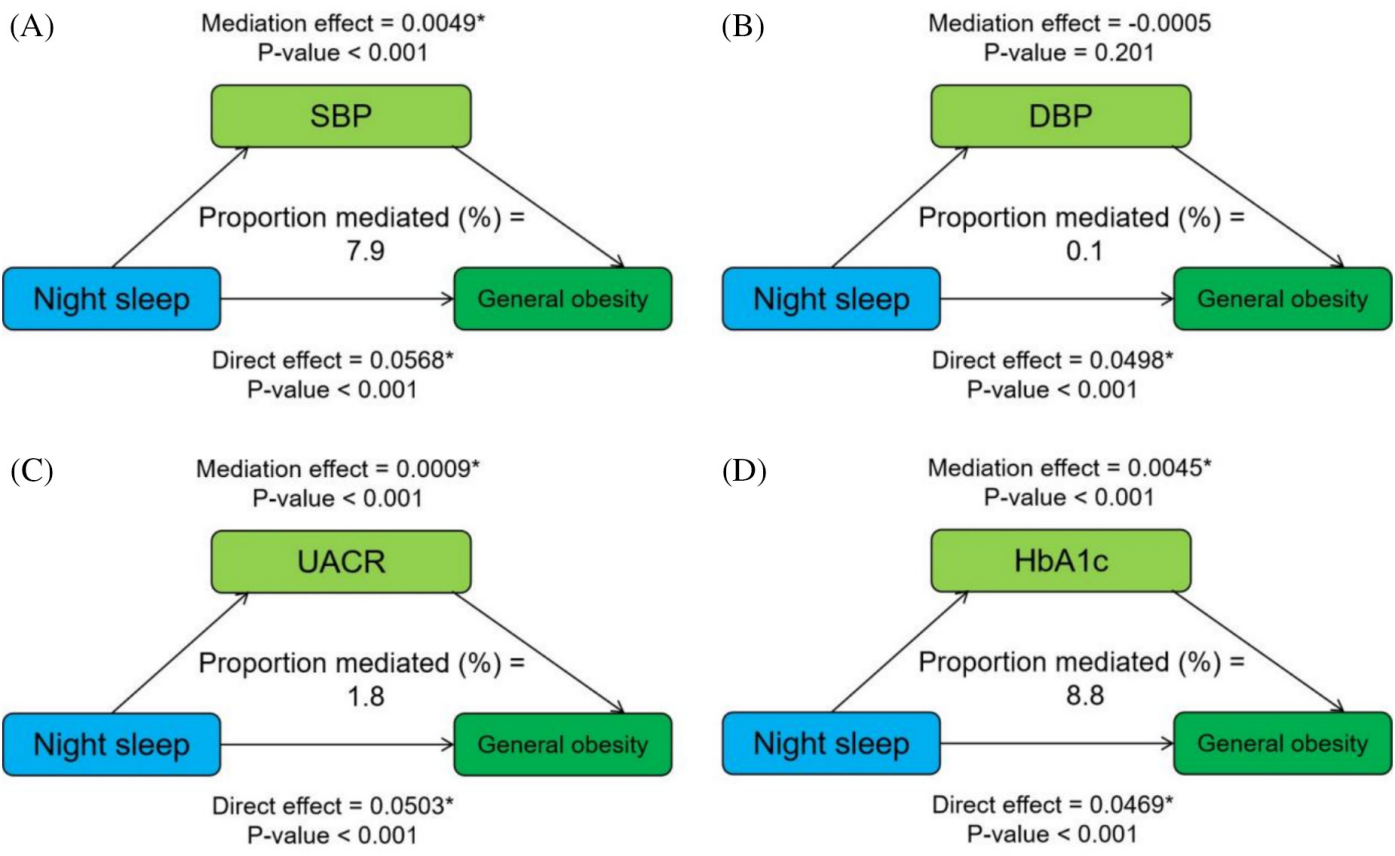
Mediation analysis of metabolic factors on the relationship between night sleep duration and general obesity.

Mediation analysis of metabolic factors on the relationship between night sleep duration and general obesity is displayed in Figure 3. SBP, UACR, and HbA1c mediated the statistical association between night sleep duration and general obesity with proportions (%) of 7.9, 1.8, and 8.8, respectively, and the mediating effect of DBP was not found to be significant. For the relationship between night sleep duration and abdominal obesity, the results showed that HbA1c mediated 15.5% of that relationship, and the remaining results were not significant. In addition, the relationship between daytime napping and general obesity was partially mediated by UACR and HbA1c, accounting for 3.2% and 13.1%, respectively (Figures S4 and S5).

## 4 Discussion

In this study, including both clinical and genetic analysis, associations between night sleep duration and daytime napping and specific obesity types and weight gain were investigated. In the clinical analysis, Cox regression results revealed a risk relationship between short sleep and general obesity, abdominal obesity, and weight gain; the results of the relationship between long sleep and obesity were not consistent, and long napping (> 1.5 h) increased the risk of general obesity rather than abdominal obesity. Metabolic factors partially explain the mechanism between night sleep, daytime napping, and obesity. In the genetic analysis among patients with T2D with medium to high genetic risk of BMI, short sleep and long napping increased the risk of general obesity compared with the low genetic risk group.

Previous studies have reported associations between different sleep phenotypes and obesity, but most of them have been reported separately, and the conclusions are still unclear or even contradictory. Meta‐regression analysis showed that short sleep duration was associated with a 38% absolute increase in the incidence of obesity and the risk of future obesity [22, 23]. Compared with intermediate (7 h) sleep duration, the risk of obesity increased 9% for each 1‐h decrease and 2% for each 1‐h increase [24]. Insufficient sleep duration was found to be associated with overweight/obesity in Greek children after adjusting for covariates [25]. Although results from epidemiological studies support the link between sleep duration and obesity, the exact mechanism of this link remains unknown. Increased appetite, craving for high carbohydrate foods, positive energy balance, and metabolic stressors may partially explain this phenomenon [26, 27, 28]. Specifically, short‐term experimental sleep restriction results for healthy adults showed an increase in subjective hunger, enhanced brain activity, and increased responsiveness to food. The concentration of plasma appetite‐related hormones changes significantly, including a decrease in leptin and an increase in auxin releasing peptide. Maintaining 4 h of sleep per night for two consecutive weeks increased the subjects' energy intake over 300 kcal/day, gained 0.5 kg of weight, and increased 11% of visceral fat [29]. It should be emphasized that there are few prospective studies that maintain short‐term experimental sleep limitations exceeding 3 weeks, so the long‐term effects of short sleep still need to be observed. In addition, previous views suggested that prolonged sleep duration in patients with chronic sleep deprivation may reverse the potential harms. However, recent studies on sleep extension on obesity and weight gain are contradictory and need to be further confirmed [30, 31, 32, 33].

Few studies exist on obesity, weight gain, and napping, especially in patients with T2D. Results of the Study of Osteoporotic Fractures showed that daytime napping was significantly related to obesity, diabetes, and Parkinson's disease, and this association was not affected by night sleep duration and fragmentation [34]. The nationwide cross‐sectional study on Saudi Arabian adolescents showed no significant association between daytime napping and metabolically healthy obesity [35]. In addition, the meta‐analysis found the significant rise in obesity occurrence when nap duration > 1 h, but no clear relationship emerged when nap duration < 1 h [36].

In our study, similar trends were also shown. Short sleep, long sleep, and long napping increased the risk of general obesity and were more pronounced in the medium to high genetic risk group defined by BMI. For abdominal obesity, follow‐up results were positive for short sleep, but not significant for long sleep, and duration of nap seems to be unrelated to it. Results of stratified analysis indicated that the above trend was more pronounced in males and patients under the age of 60. In addition, both short and long sleep increased the risk of weight gain, regardless of using BMI or VFA as the evaluation criteria, but excessively long naps only increased the risk of weight gain defined by BMI and were not related to changes in visceral fat.

### 4.1 Strengths and Limitations

Key strengths of this study are the following: (1) our study systematically focused on the association between sleep phenotypes and obesity types, weight gain among Chinese patients with T2D, and it was the largest research in the relevant field according to our knowledge. (2) Our study not only involved clinical analysis but also discussed the differentiated performance of genetic risk of BMI‐defined obesity, which was more in line with the concept of precision medicine. In addition, our study has a few limitations. First, although the results of this study suggest that short sleep and long napping increased the risk of general obesity, they do not involve causal associations and it is currently unclear whether short sleep leads to obesity or whether obesity leads to insufficient sleep, or both. Second, the study population is patients with T2D in East Asia, and whether it can be extended to the non‐East Asian populations or individuals without diabetes still needs further verification, so the conclusions should be interpreted with caution. Third, the sleep phenotype grouping used in this article was based on participants' questionnaires at baseline, which were self‐reported by patients and may have recall bias and changes in relevant sleep behavior during subsequent follow‐up. Considering the randomness and noninterference of this issue, we believe that such error would be expected to bias toward the null, although this would lead to an underestimation of the risk estimates. Fourthly, due to the limitations of real‐world studies, the follow‐up analysis was only limited to a portion of patients, which may bias the relevant study findings. We will expand the number of follow‐up visits to mitigate this potential confounding impact in further research. Although the mediation model used in our study assumes a linear relationship between variables, there may be uncaught nonlinear dynamics in the interaction between sleep, metabolic factors, and obesity. For example, the negative impact of poor sleep on metabolic abnormalities may significantly increase after a specific threshold, or there may be heterogeneity in mediating pathways among different genetic risk subgroups. Future research can use piecewise linear models, machine learning‐driven mediation analysis (such as gradient boosting mediation models), or nonlinear structural equation models to further explore such complex relationships. In addition, although we have adjusted for several covariates, there are potential confounders that could influence the results, such as dietary habits, physical activity, socioeconomic biases, and glucose‐lowering medications, which we did not include in this study, and further research is needed to strengthen our understanding of these complex associations.

## 5 Conclusions

In summary, our findings suggest that short sleep, long sleep, and long napping increased the risk of general obesity and BMI‐defined weight gain, and were more pronounced in the medium to high genetic risk group. Duration of nap seems to be unrelated to abdominal obesity and VFA‐defined weight gain among patients with T2D. Metabolic factors partially explain the mechanism between sleep and obesity. Our study could help physicians to more accurately guide patients in lifestyle adjustments and improve long‐term outcomes.

## Author Contributions

Guang Ning, Yifei Zhang, Yanan Cao, and Weiqing Wang contributed to the conception and design of the study. Li Li, Songbo Fu, Yuancheng Dai, Juan Shi, Yanmei Yu, Ying Peng, Hongmei Qiu, Jinsong Kuang, Hongyun Lu, Huige Shao, Chunlei Yuan, Xiaohu Wang, Ping Zhang, Sheli Li, Yanhui Pan, Ling Hu, Zhigang Zhao, Yunxia Chen, Jian Kuang, Yi Shu, Jinhua Qian, Qibin Mao, Jieji Zhang, Yan Liu, Hong Yang, Zhaoli Yan, Weici Xie, Qian Zhang, Ping Zhang, Hongji Wu, Ling Gao, Yongjun Jin, Ning Xu, Chaoyang Xu, Yifei Zhang, and Weiqing Wang contributed to data collection and patient care. Lei Xi, Xiaohui Sun, Zhimin Feng, Qing Zhang and Lin Li contributed to the analysis of data. Lei Xi, Li Li, Songbo Fu, Yuancheng Dai, Juan Shi, and Yanmei Yu interpreted the results and wrote the initial manuscript. All authors contributed to data interpretation, draft revision and approval of the final manuscript. Yifei Zhang, Yanan Cao, and Weiqing Wang contributed to the study supervision and are the guarantors of this work. All authors have reviewed and approved the final manuscript.

## Conflicts of Interest

The authors declare no conflicts of interest.

## Supporting information


**Data S1.** Supporting Information.

## Data Availability

The datasets used and/or analyzed during the current study are available from the corresponding author on reasonable request.
