# Supplementary material for: Sleep Phenotypes, Genetic Susceptibility, and Risk of Obesity in Patients With Type 2 Diabetes: A National Prospective Cohort Study
Source: J Diabetes. 2025 May 20;17(5):e70095. doi: 10.1111/1753-0407.70095 (PMC12092374; doi:10.1111/1753-0407.70095)
Supplement: Supplementary file 1 — Data S1. Supporting Information. [file JDB-17-e70095-s001.docx]

**Supplementary materials**


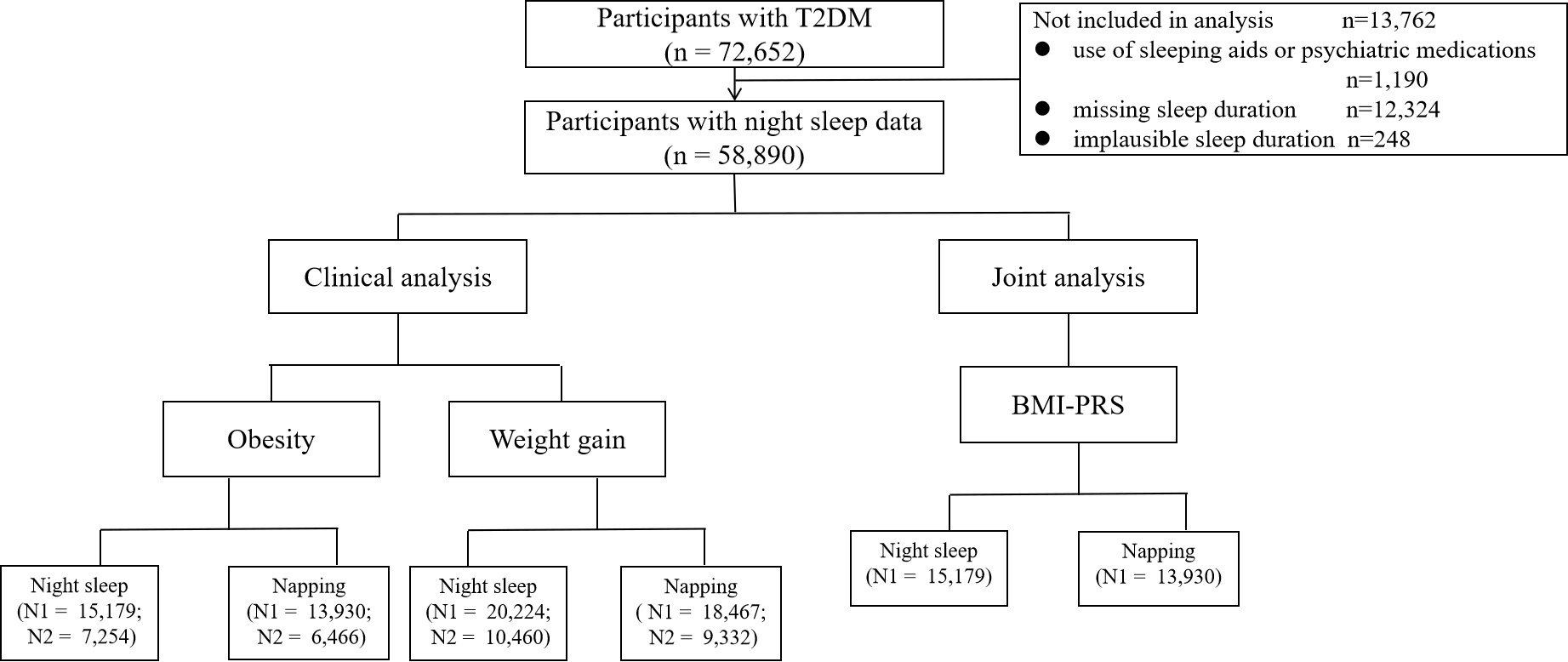


**Figure S1: Study population and flowchart of the participants included in final analyses.** N1, Number of participants included when the variable was BMI; N2, Number of participants included when the variable was VFA. BMI, body mass index; VFA, visceral fat area.


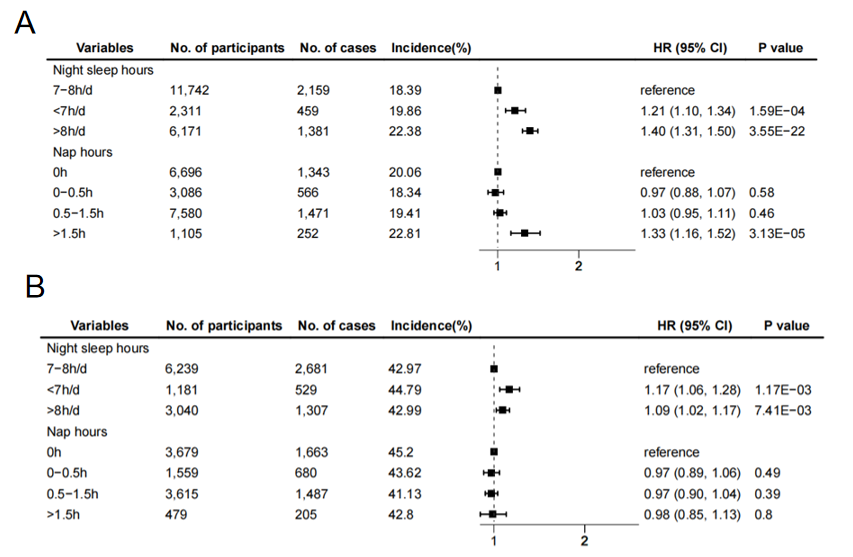


**Figure S2. Associations of different sleep phenotypes with weight gain among patients with type 2 diabetes in minimally adjusted model.** Weight gain was defined as an increase in BMI (A) or VFA (B) by more than 5%. Model was adjusted for age and sex.


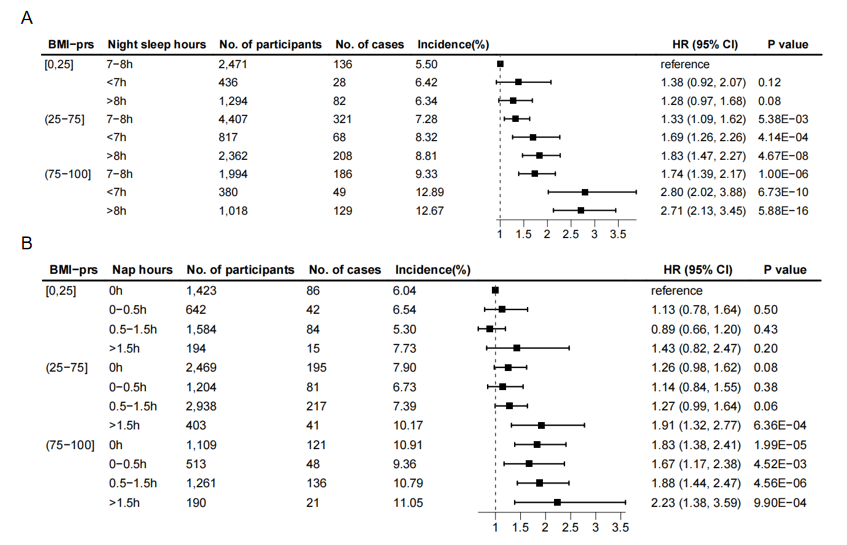


**Figure S3. The joint association of night sleep duration, nap duration and genetic risk of BMI with general obesity.** BMI-PRS, polygenic risk score for body mass index and scores were ranked from low to high. Model was adjusted for age and sex.


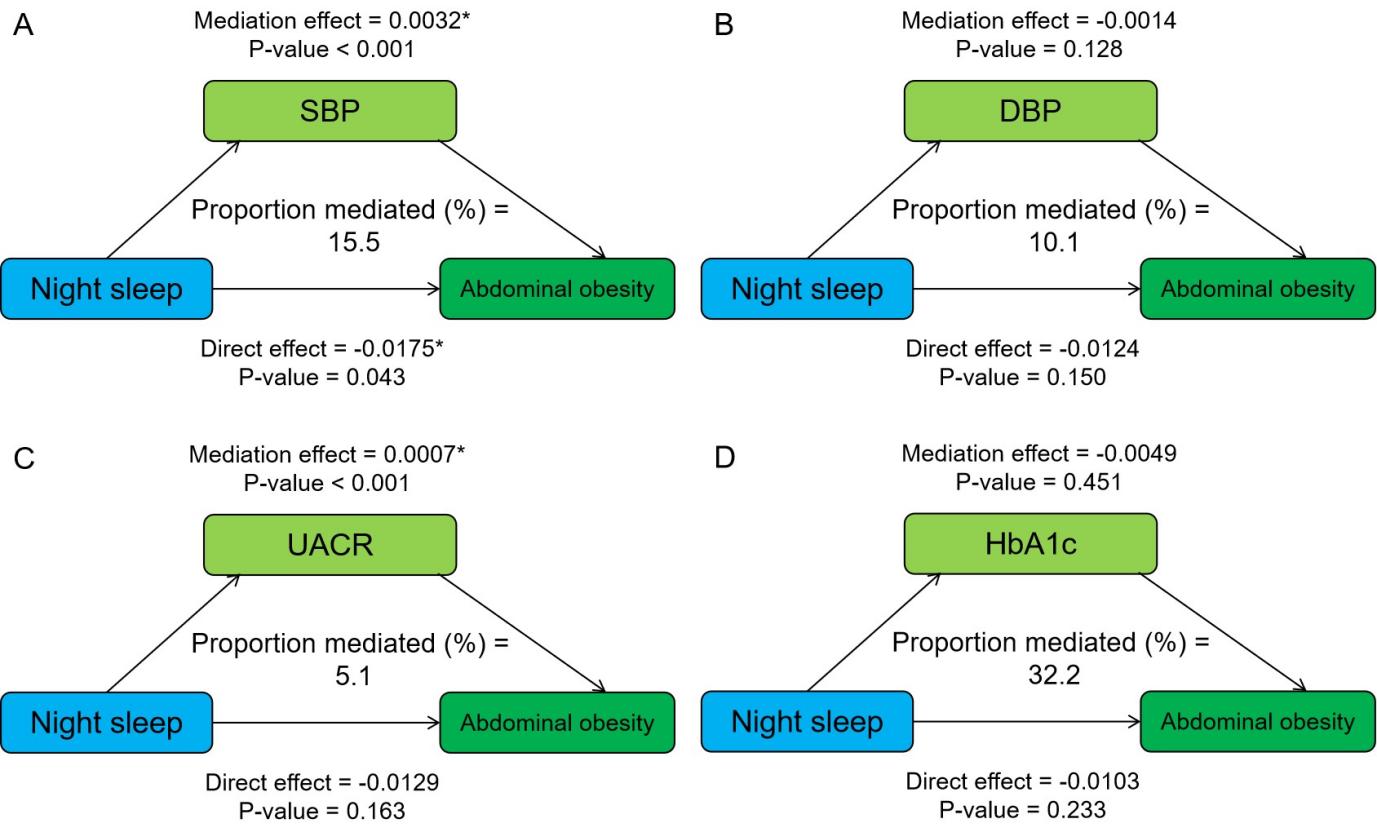


**Figure S4. Mediation analysis of metabolic factors on the relationship between night sleep duration and abdominal obesity**.


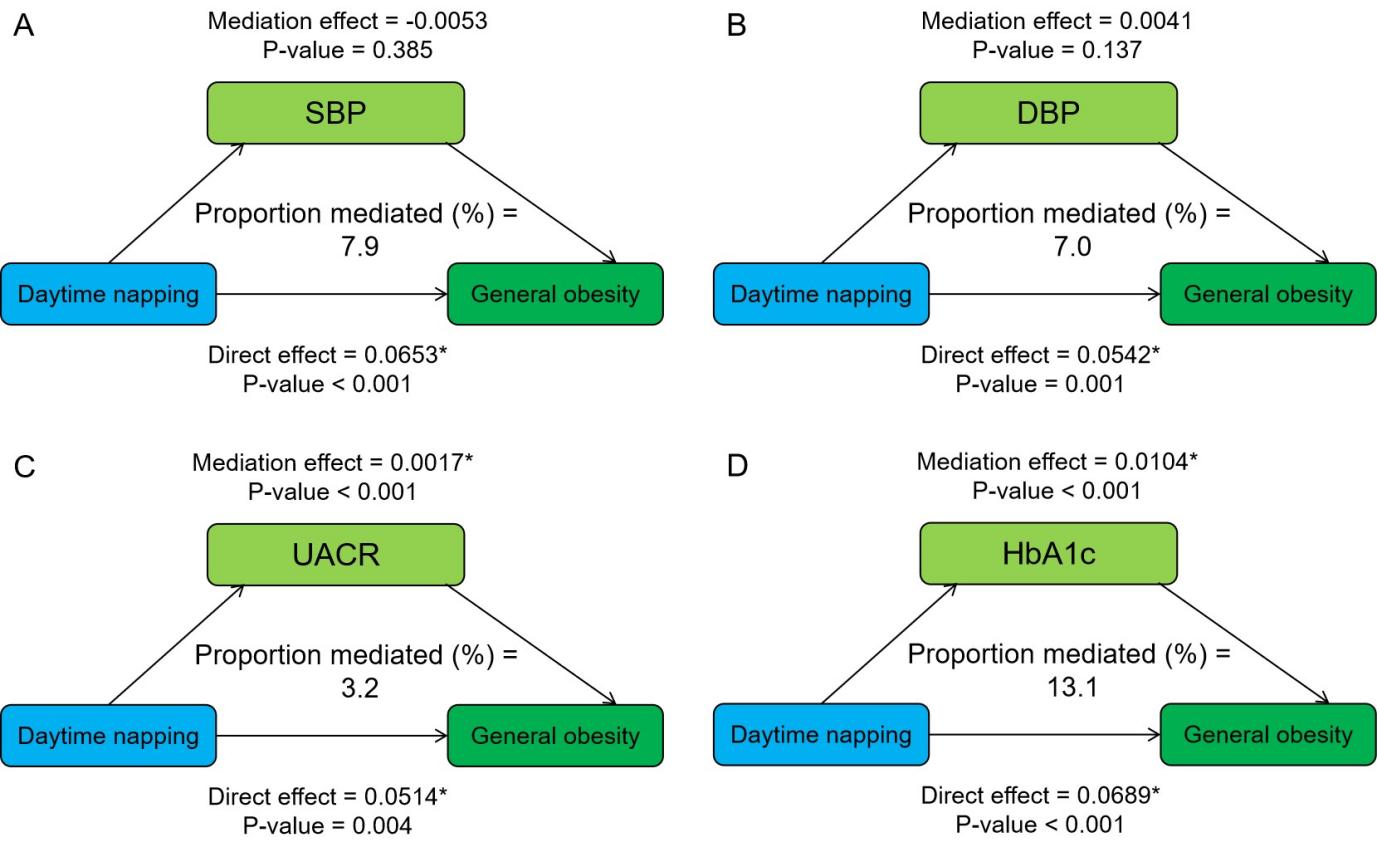


**Figure S5. Mediation analysis of metabolic factors on the relationship between daytime napping and general obesity**.

**Table S1 Detailed description of the public BMI-PRS model**

| Database Fields | detailed information |
| --- | --- |
| PGS Catalog ID | PGS002360 |
| Traits in PGS Catalog | body mass index |
| Development Sample Numbers | 124,000 individuals |
| Development Sample Ancestry | East Asian |
| Development Method | BOLT-LMM-BBJ |
| Cohort | Biobank Japan |
| Performance Metrics | 916 East Asian; R^2^:0.0397 |
| Number of Variants | 920,920 |
| Effect Weight Type | beta |

**Table S2 Cox Regression Analyses for Associations of night sleep duration With General Obesity and Abdominal Obesity in Participants by Sex or Age**

|  | General obesity | | Abdominal obesity | |
| --- | --- | --- | --- | --- |
| Variables | HR (95% CI) | *P* Value | HR (95% CI) | *P* Value |
| Age, y |  |  |  |  |
| ≥60 |  |  |  |  |
| Short sleep | 1.33 (0.96-1.85) | 0.09 | 1.14 (0.80-1.63) | 0.46 |
| Long sleep | 1.43 (1.16-1.76) | <0.01 | 1.23 (0.98-1.54) | 0.07 |
| <60 |  |  |  |  |
| Short sleep | 1.43 (1.14-1.80) | <0.01 | 1.46 (1.13-1.90) | <0.01 |
| Long sleep | 1.30 (1.11-1.53) | <0.01 | 1.02 (0.83-1.25) | 0.88 |
| Sex |  |  |  |  |
| Men |  |  |  |  |
| Short sleep | 1.47 (1.16-1.85) | <0.01 | 1.20 (0.92-1.56) | 0.18 |
| Long sleep | 1.26 (1.06-1.49) | <0.01 | 1.07 (0.89-1.29) | 0.47 |
| Women |  |  |  |  |
| Short sleep | 1.33 (0.97-1.83) | 0.08 | 1.64 (1.15-2.33) | <0.01 |
| Long sleep | 1.50 (1.23-1.83) | <0.01 | 1.20 (0.92-1.55) | 0.17 |

**Table S3 Cox Regression Analyses for Associations of Daytime Napping With General Obesity and Abdominal Obesity in Participants by Sex or Age**

|  | General obesity | | Abdominal obesity | |
| --- | --- | --- | --- | --- |
| Variables | HR (95% CI) | *P* Value | HR (95% CI) | *P* Value |
| Age, y |  |  |  |  |
| ≥60 |  |  |  |  |
| >0-0.5 | 0.84 (0.61-1.17) | 0.31 | 0.96 (0.70-1.31) | 0.79 |
| >0.5-1.5 | 1.02 (0.80-1.29) | 0.89 | 1.04 (0.81-1.33) | 0.76 |
| >1.5 | 1.30 (0.85-2.00) | 0.22 | 1.11 (0.69-1.78) | 0.67 |
| <60 |  |  |  |  |
| >0-0.5 | 1.08 (0.86-1.36) | 0.49 | 0.96 (0.74-1.26) | 0.79 |
| >0.5-1.5 | 0.98 (0.82-1.17) | 0.83 | 0.97 (0.78-1.19) | 0.75 |
| >1.5 | 1.42 (1.04-1.94) | 0.03 | 1.22 (0.82-1.83) | 0.33 |
| Sex |  |  |  |  |
| Men |  |  |  |  |
| >0-0.5 | 1.16 (0.92-1.46) | 0.21 | 0.99 (0.77-1.27) | 0.93 |
| >0.5-1.5 | 0.99 (0.82-1.18) | 0.88 | 0.99 (0.81-1.20) | 0.91 |
| >1.5 | 1.56 (1.14-2.14) | <0.01 | 1.08 (0.72-1.62) | 0.70 |
| Women |  |  |  |  |
| >0-0.5 | 0.77 (0.56-1.07) | 0.12 | 0.84 (0.59-1.20) | 0.33 |
| >0.5-1.5 | 1.04 (0.83-1.30) | 0.74 | 0.96 (0.73-1.27) | 0.77 |
| >1.5 | 1.14 (0.75-1.73) | 0.54 | 1.35 (0.84-2.18) | 0.22 |

**Table S4 Associations of different sleep phenotypes with obesity types, adjusted for age and sex**

|  | General obesity | | Abdominal obesity | |
| --- | --- | --- | --- | --- |
| Variables | HR (95% CI) | *P* Value | HR (95% CI) | *P* Value |
| Night sleep duration |  |  |  |  |
| Intermediate sleep |  |  |  |  |
| Short sleep | 1.40 (1.17-1.68) | <0.01 | 1.33 (1.09-1.61) | <0.01 |
| Long sleep | 1.40 (1.24-1.58) | <0.01 | 1.12 (0.97-1.30) | 0.11 |
| Daytime Napping |  |  |  |  |
| None |  |  |  |  |
| >0-0.5 | 0.96 (0.80-1.15) | 0.64 | 0.89 (0.74-1.08) | 0.24 |
| >0.5-1.5 | 0.99 (0.86-1.14) | 0.90 | 0.92 (0.79-1.07) | 0.27 |
| >1.5 | 1.42 (1.12-1.82) | <0.01 | 1.10 (0.82-1.48) | 0.54 |

**Supplementary Information**

**Participating members of the MMC**

1. Tingyu Ke (The Second Affiliated Hospital of Kunming Medical University, Kunming, China),

2. Dong Zhao (Beijing Luhe Hospital, Capital Medical University, Beijing Key Laboratory of Diabetes Research and Care, Beijing, China),

3. Bangqun Ji (Xingyi People's Hospital, Xingyi, China),

4. Sunjie Yan (The First Affiliated Hospital, Fujian Medical University, Fujian Province, China),

5. Yinghong Du (Guangzhou Panyu Central Hospital, Guangzhou, China),

6. Fengmei Xu (General Hospital of Hebi Coal Industry (Group) Co., Ltd., Hebi, China),

7. Xuejiang Gu (The First Affiliated Hospital of Wenzhou Medical University, Wenzhou, China),

8. Weijiang Chu (Laizhou People's Hospital, Laizhou, China),

9. Yufan Wang (Shanghai General Hospital, Shanghai Jiao Tong University School of Medicine, Shanghai, China),

10. Liujun Fu (The First Affiliated Hospital, College of Clinical Medicine, Henan University of Science and Technology, Luoyang, China),

11. Yikui Lv (The People's Hospital of Longkou, Shandong Province, China),

12. Yan Wang (The First People's Hospital of Yunnan Province, Kunming, China),

13. Zunhai Zhou (Yangpu Hospital, Tongji University School of Medicine, Shanghai, China),

14. Yuqin Zeng (Affiliated Taihe Hospital of Hubei University of Medicine, Shiyan, China),

15. Qijuan Dong (People's Hospital of Zhengzhou, Zhengzhou, China),

16. Mao Ye (The Central Hospital of Enshi Tujia and Miao Autonomous Prefecture, Enshi Clinical College of Wuhan University, Enshi, China),

17. Shu Li (Huizhou Municipal Central Hospital, Huizhou, China),

18. Guixia Wang (The First Hospital of Jilin University, Changchun, China),

19. Yu Shi (Qidong People's Hospital, Qidong Liver Cancer Institute, Affiliated Qidong Hospital of Nantong University, Qidong, China),

20. Yawei Zhang (Pingxiang People's Hospital, Pingxiang, China),

21. Lei Chen (Suzhou Municipal Hospital Affiliated to Nanjing Medical University, Suzhou, China),

22. Rongyue Chen (Xuchang Hongyue diabetes Hospital, Xuchang, China),

23. Qiaoying You (Shaoxing People's Hospital, Shaoxing, China),

24. Riqiu Chen (Lishui People's Hospital, Lishui, China),

25. Ling Yang (Affiliated Hospital of Jiangsu University, Zhenjiang, China),

26. Xiaohua Yang (Hai'an People's Hospital, Jiangsu Province, China),

27. Wen Xu (Jiangyin Harbour Hospital, Jiangsu Province, China),

28. Haibing Chen (Shanghai Tenth People's Hospital, School of Medicine Tongji University, Shanghai, China),

29. Qidong Zheng (The Second People's Hospital of Yuhuan, Yuhuan, China),

30. Chenzhong Li (Third Affiliated Hospital of Southern Medical University, Guangdong Province, China),

31. Xingjian Zhou (Xiangyang First People's Hospital, Affiliated to Hubei Medical College, Hubei Province, China),

32. Qin Wan (Affiliated Hospital of Southwest Medical University, Sichuan Province, China),

33. Mingjun Gu (The Shanghai Pudong New Area Gongli Hospital, Shanghai, China),

34. Libo Chen (Shenzhen Nanshan Hospital, Shenzhen, China),

35. Shuzeng Cheng (Xin Mi Hospital of Traditional Chinese Medicine, Henan Province, China),

36. Lin Zhang (Bayannur Hospital, Inner Mongolia Autonomous Region, China),

37. Yinghui Yang (The First Affiliated Hospital of Qiqihar Medical College, Heilongjiang Province, China),

38. Yufan Wang,Yongde Peng (Shanghai General Hospital, Shanghai Jiao Tong University School of Medicine, Shanghai, China),

39. Xing Liu (Ansteel Group Hospital, Liaoning Province, China),

40. Mingfu Ma (The Fifth Pople's Hospital of Qinghai Province , Qinghai Province, China),

41. Xinrong Xie (Minzu Hospital of Guangxi Zhuang Autonomous Region, Guangxi, China),

42. Weiping Tu (Shangyu People's Hospital of Shaoxing City, Zhejiang Province, China),

43. Yanfang Zhang (Luoyang Central Hospital Affiliated to Zhengzhou University, Henan Province, China),

44. Tianshu Gao (The Affiliated Hosptial of Liaoning University of Traditional Chinese Medicine, Liaoning Province, China),

45. Xinghai Zhao (Kunshan Hospital of Traditional Chinese Medicine, Jiangsu Province, China),

46. Ming Li (Chengdu Ruien Diabetes Hospital, Sichuan Province, China),

47. Xin Yan (The Second People's Hospital of Nanning City, Guangxi, China),

48. Hailin Shao (Tianjin Fourth Central Hospital, Tianjin, China),

49. Dongxue Su (Heilongjiang agricultural reclamation construction sanjiang people's hospital, Heilongjiang Province, China),

50. Hui Guo (The First Affiliated Hospital of Xi'an JiaoTong University, Shaanxi Province, China),

51. Jing Bai (Gongyi City People's Hospital, Henan Province, China),

52. Hongxia Liu (Guangdong Provincial People's Hospital Nanhai Hospital, Guangdong Province, China),

53. Yubo Sha (The First People's Hospital of Dali, Yunnan Province, China),

54. Libin Liu (Fujian Medical University Union Hospital, Fujian Province, China),

55. Pei Gu (Sinopharm Tongmei General Hospital, Shanxi Province, China),

56. Peng Yang (Shaanxi diabetes Hospital, Shaanxi Province, China),

57. Hongyan Deng (Wuhan Fourth Hospital, Hubei Province, China),

58. Feng Wei (The First Affiliated Hospital of Baotou Medical College,Inner Mongolia University of Science and Technology, Inner Mongolia Autonomous Region, China),

59. Xueyong Lou (Jinhua Central Hospital, Zhejiang Province, China),

60. Hongmei Wu (Longgang Central Hospital of Shenzhen, Shenzhen, China),

61. Ping Liu (General Hospital of Ningxia Medical University, Ningxia Hui Autonomous Region, China),

62. Ying Shen (Anyang Hospital of Traditional Chinese Medicine , Henan Province, China),

63. Yao Li (The First Affiliated Hospital of Chengdu Medical College, Sichuan Province, China),

64. Xia Jiang (Tianjin First Central Hospital, Tianjin, China),

65. Dongmei Li (People's Hospital of Inner Mongolia Autonomous Region, Inner Mongolia Autonomous Region, China),

66. Xueqin Wang (First People's Hospital of Nantong, Jiangsu Province, China),

67. Yingfen Qin (The First Affiliated Hospital of Guangxi Medical University, Guangxi, China),

68. Yong Chen (Central Hospital of Eastern Hubei Medical Group, Hubei Province, China),

69. Yanhong Gao (The Fifth Pople's Hospital of Datong, Shanxi Province, China),

70. Mianjiong Wang (Shantou Chenghai District People's Hospital, Guangdong Province, China),

71. Guoqing Liu (Shengli Oilfield Central Hospital, Province, China),

72. Xiaomin Xie (The First People's Hospital of Yinchuan, Ningxia Hui Autonomous Region, China),

73. Zhiqiang Kang (ZhengZhou Central Hospital, Henan Province, China),

74. Lianwei Wang (Zhumadian Central Hospital, Henan Province, China),

75. Lijun Wang (Taizhou First People's Hospital, Zhejiang Province, China),

76. Liu Yang (Jingjiang People's Hospital in Jiangsu Province, Jiangsu Province, China),

77. Xinhong Lu (Nanxishan Hospital of Guangxi Zhuang Auton-omous Region, Guangxi, China),

78. Zhanpeng He (Liwan Central Hospital, Guangdong Province, China),

79. Jiansheng Niu (Yulin Second Hospital, Shanxi Province, China),

80. Zhihua Jiang (Suzhou Jinchang Hospital, Jiangsu Province, China),

81. Lin Wu (The People's Hospital of Nanpi, Hebei Province, China),

82. Jing Wang (Weifang Municipal Hospital, Shandong Province, China),

83. Jianling Du (The First Affiliated Hospital of Dalian Medical University, Liaoning Province, China),

84. Xin Liao (Affiliated Hospital of Zunyi Medical College, Guizhou Province, China),

85. Shuhui Yang (Shantou Central Hospital, Guangdong Province, China),

86. Yezi Sun (Zhangjiagang First People's Hospital, Jiangsu Province, China),

87. Yiyuan Yao (First People's Hospital of Xiushui County , Jiangxi Province, China),

88. Huimin Zhou (The First Hospital of Hebei Medical University, Hebei Province, China),

89. Zhongyan Shan (The First Affiliated Hospital of China Medical University, Liaoning Province, China),

90. Qian Xiong (Shanghai Gonghui Hospital, Shanghai, China),

91. Guiyan Chen (Binzhou People's Hospital , Shandong Province, China),

92. Chunli Piao (Shenzhen Hospital (Futian), Guangzhou University of Chinese Medicine, Shenzhen, China),

93. Li Qin (Xinhua hospital Chongming branch, Shanghai, China),

94. Lin Tian (The Ninth People's Hospital of Zhengzhou, Henan Province, China),

95. Xiaoqian Wang (People's Hospital of Deyang City, Sichuan Province, China),

96. Hanmin Wang (The First People's Hospital of Qujing City, Yunnan Province, China),

97. Yi Peng (Zhangjiakou First Hospital, Hebei Province, China),

98. Dadong Fei (Zaozhuang Municipal Hospital, Shandong Province, China),

99. Yinli Huang (The Second People's Hospital of Pingyang County, Zhejiang Province, China),

100. Jie Han (Hebei Petro China Central Hospital, Hebei Province, China),

101. Binhong Duan (Heilongjiang Provincial Hospital, Heilongjiang Province, China),

102. Xiaomin Wang (The First People's Hospital of Baiyin, Gansu Province, China),

103. Xiaolin Ye (The First People's Hospital of Tonglu，Zhejiang Chinese Medical University, Zhejiang Province, China),

104. Binbin Tian (Yuzhou City People's Hospital, Henan Province, China),

105. Zhigang Wen (Jiangmen Central Hospital, Jianghai Branch, Guangdong Province, China),

106. Yuhong Chen (Ruijin Hospital, Shanghai Jiao Tong University School of Medicine, Shanghai, China),

107. Lu Lu (First Hospital of Qinhuangdao, Hebei Province, China),

108. Fan Zhang (Peking University Shenzhen Hospital, Shenzhen, China),

109. Ping Tang (Shenzhen Luohu Hospital Group, Shenzhen, China),

110. Zuhua Gao (Taizhou Hospital of Zhejiang Province, Wenzhou Medical University, Zhejiang Province, China),

111. Wei Wang (Union Hospital, Tongji Medical College, Huazhong University of Science and Technology, Hubei Province, China),

112. Ping Liu (Northwest University Affiliated Hospital,Xi'an No.3 Hospital, Shaanxi Province, China),

113. Zhihong Zeng (Longquan People's Hospital, Zhejiang Province, China),

114. Dongfang Liu (the Second Affiliated Hospital of Chongqing Medical University, Chongqing, China),

115. Guoxi Jin (The First Affiliated Hospital of Bengbu Medical College, Anhui Province, China),

116. Xiaoshu Wang (West China-Guang'an Hospital, Sichuan University, Sichuan Province, China),

117. Xinhua Ye (Changzhou No.2 People's Hospital, Jiangsu Province, China),

118. Yugang Hu (Chaozhou People's Hospital, Guangdong Province, China),

119. Ping Xiong (Affiliated Hospital of Chengdu University, Sichuan Province, China),

120. Pin Chen (The 900th Hospital of the Joint Logistics Support Force of the Chinese People's Liberation Army, Fujian Province, China),

121. Weiming Wu (Changshu Hospital of Traditional Chinese Medicine, Jiangsu Province, China),

122. Yunjuan Gu (Affiliated Hospital of Natong University, Jiangsu Province, China),

123. Wei Tang (Geriatric Hospital of Nanjing Medical University, Jiangsu Province, China),

124. Xuemei Yu (Shanghai Fengxian District Central Hospital, Shanghai, China),

125. Zhen Zhang (First People's Hospital of Shangqiu , Henan Province, China),

126. Zhenfeng Shi (Xingtai People's Hospital, Hebei Province, China),

127. Wei Wang (Xiang'an Hospital of Xiamen University, Fujian Province, China),

128. Shuibing Yang (The First People's Hospital of Huaihua, Hunan Province, China),

129. Qirong Gao (Chongzhou People's Hospital, Sichuan Province, China),

130. Chunxiao Shi (Anshun People's Hospital, Guizhou Province, China),

131. Yuchun Liu (Taikang County People's Hospital, Henan Province, China),

132. Yan Yang (Sichuan Academy of Medical Sciences, Sichuan Provincial People's Hospital, Sichuan Province, China),

133. Jianfeng Liu (Cangzhou People's Hospital (Oncology Area), Hebei Province, China),

134. Mingjuan Luo (The University of Hong Kong-Shenzhen Hospital, Shenzhen, China),

135. Changxiu Guo (Hongqi Hospital Affiliated to Mudanjiang Medical University , Heilongjiang Province, China),

136. Lingling Xu (Shenzhen Hospital of Southern Medical University, Shenzhen, China),

137. Yachao Yang (Weihai Municipal Hospital, Shandong Province, China),

138. Xiaohua Li (Shanghai Seventh People's Hospital , Shanghai, China),

139. Xiuwei Zhang (Dongguan People's Hospital, Guangdong Province, China),

140. Shengli Wu (Endocrine and Metabolic Center, Karamay Hospital of traditional Chinese and Western medicine (Karamay People's Hospital), Karamay, China),

141. Hongjun Fu (Luqiao Hospital of Taizhou Enze Medical Center (Group), Zhejiang Province, China),

142. Yan Feng (Mudanjiang City Second People's Hospital, Heilongjiang Province, China),

143. Qun Liu (PKUCare Luzhong Hospital, Shandong Province, China),

144. Huiju Zhong (Xiangya Changde Hospital, Hunan Province, China),

145. Aixue Wang (The People's Hospital of Yongcheng, Henan Province, China),

146. Yu Zhao (Bao'an District Central Hospital, Shenzhen, China),

147. Ciyou Huang (Second People's Hospital of Wuxi, Jiangsu Province, China),

148. Wangjun Chen (Taicang Shaxi people's Hospital , Jiangsu Province, China),

149. Li Yan (Sun Yatsen Memorial Hospital, Sun Yat-sen University, Guangdong Province, China),

150. Ming Niu (Nanxiang Town Community Health Service Center, Jiading District, Shanghai, China),

151. Chuanqi Chen (Shekou People's Hospital,Nanshan District, Shenzhen City, Shenzhen, China),

152. Yaoming Xue (Nanfang Hospital, Southern Medical University, Guangdong Province, China),

153. Hongxia Zhao (The People's Hospital of Linyi County, Shandong Province, China),

154. Peng Duan (The Third Hospital of Nanchang, Jiangxi Province, China),

155. Xuejian Ni (Taiping Street Community Health Service Center, Xiangcheng District, Jiangsu Province, China),

156. Ying Yang (Affiliated Hospital of Yunnan University,the Second People's Hospital of Yunnan Province, Yunnan Province, China),

157. Hongcheng Ding (Shiyan People's Hospital, Hubei Province, China),

158. Lianhuan Zhang (Shaoxing Hospital of Traditional Chinese Medicine, Zhejiang Province, China),

159. Qiaoyun Qian (Dongtai Hospital of Traditional Chinese Medicine, Jiangsu Province, China),

160. Xinguo Hou (Qilu Hospital,Shandong University, Shandong Province, China),

161. Lan Xu (Wuxi People's Hospital, Jiangsu Province, China),

162. Zhengyi Tang (Wuxi Branch of Ruijin Hospital Affiliated to Medical College of Shanghai Jiao Tong University, Jiangsu Province, China),

163. Yuanyuan Lin (The First People's Hospital of Nanning, Guangxi, China),

164. Chunfang Qian (Chedun Town Community Health Service Center, Songjiang District, Shanghai, China),

165. Yanhong Zhao (Fenghua People's Hospital, Zhejiang Province, China),

166. Yuelin Li (People's Hospital of Xinping Yi and Dai Autonomous County, Yunnan Province, China),

167. Hongyan Shu (Linzi District People's Hospital of Zibo City, Shandong Province, China).
